# Assessment of Purslane (*Portulaca oleracea* L.) Total Oxalate Content, Ascorbic Acid, and Total Organic Acids Using Near-Infrared Spectroscopy

**DOI:** 10.3390/plants14223426

**Published:** 2025-11-09

**Authors:** Zornitsa Zherkova, Mima Todorova, Neli Grozeva, Milena Tzanova, Antoniya Petrova, Petya Veleva, Stefka Atanassova

**Affiliations:** 1 Department of Biological Sciences, Faculty of Agriculture, Trakia University, 6000 Stara Zagora, Bulgaria; n.grozeva@trakia-uni.bg (N.G.); milena.tsanova@trakia-uni.bg (M.T.); 2 Department of Plant Breeding, Faculty of Agriculture, Trakia University, 6000 Stara Zagora, Bulgaria; mima.todorova@trakia-uni.bg; 3 Department of Agricultural Engineering, Faculty of Agriculture, Trakia University, 6000 Stara Zagora, Bulgaria; antoniya.petrova@trakia-uni.bg (A.P.); petya.veleva@trakia-uni.bg (P.V.); stefka.atanasova@trakia-uni.bg (S.A.)

**Keywords:** ascorbic acid, near-infrared spectroscopy, non-destructive methods, oxalate content, *Portulaca oleracea* L., processing treatments, purslane, spectrophotometry, total organic acids

## Abstract

Purslane (*Portulaca oleracea* L.) has been regaining its reputation as a valuable food and source of nutrients and biologically active compounds, but a high content of oxalates reduces mineral bioavailability and poses nutritional limitations. This study evaluated the influence of culinary processing on oxalate content in purslane and the potential of near-infrared (NIR) spectroscopy for non-destructive assessment of total oxalates, ascorbic acid, and total organic acid. The ascorbic acid and total organic acid in fresh samples, and the total oxalate content of fresh, blanched, and pickled samples were determined. Culinary treatments (blanching and pickling) reduced oxalate content. The highest oxalate content was observed in fresh samples (33.38–61.84 g/100 g), lower in blanched samples (19.07–34.36 g/100 g), and the lowest content in pickling samples (10.48–18.31 g/100 g). NIR spectra (900–1700 nm) of the analyzed samples were measured, and PLS regression was used for the determination of tested components. The NIR spectroscopy achieved high predictive accuracy for ascorbic acid, total organic acid, and oxalate content. Rcval > 0.98 and SECV values between 0.02 and 0.38 g/100 g for oxalate content. NIR spectroscopy provides a rapid, accurate, and non-destructive alternative to conventional methods for oxalate determination in fresh, blanched, and pickled plant tissues, ascorbic acid and organic acid in fresh samples.

## 1. Introduction

Purslane (*Portulaca oleracea* L.) is an annual herbaceous plant of the family Portulacaceae, widely distributed in temperate and tropical climate zones worldwide. *Portulaca oleracea* is sometimes considered and treated as a weed due to its rapid growth patterns and ability to adapt to different conditions. In recent decades, purslane has been gaining attention and regaining its reputation as a valuable food and medicinal plant, much like it was known in the past. Common purslane is included in the list of medicinal plants according to the Medicinal Plants Act in Bulgaria [1]. In Bulgaria, it is found as a ruderal plant up to approximately 1000 m above sea level, in deserted areas, as a weed in gardens, along roads, fences, sidewalks, and anywhere near places affected by anthropogenic activity [2,3,4]. Purslane has an impressive nutritional profile—a rich plant source of omega-3 fatty acids (α-linolenic acid), vitamins (ascorbic acid, α-tocopherol, β-carotene) [5,6,7], flavonoids [8], has a high protein and mineral content, and a low fat content [9], which defines *P. oleracea* as a plant with potential for use in dietary and preventive nutrition.

Despite its numerous benefits and nutritional value, purslane also contains antinutrients, among which the most important are oxalates. Oxalic acid can bind to minerals (such as Ca, Fe, and Mg) and form both soluble and insoluble salts, thereby preventing their absorption by the body [10]. Oxalic acid can form insoluble calcium oxalate crystals by binding with calcium, which is associated with a risk of kidney stone formation and other metabolic disorders [11]. A quantitative assessment of oxalate in purslane is important, especially when consumed fresh or as part of a specialized diet. Oxalates are end metabolites of many plant species. They are found in varying concentrations in plant tissues, especially in leafy, root vegetables, and beans. Spinach (*Spinacia oleracea* L.) and soybeans (*Glycine max* (L.) Merr) are well known for their high oxalate content [12,13]. Representatives of the families Oxalidaceae—sorrel (*Oxalis* spp.), Polygonaceae—duckweed (*Rumex* spp.), rhubarb (*Rheum* spp.), buckwheat (*Fagopyrum* spp.), Amaranthaceae—*S. oleracea*, beetroot (*Beta vulgaris* L.), orach (*Atriplex* spp.), Convolvulaceae—sweet potato (*Ipomoea batatas* L.), Portulacaceae—*P. oleracea*, Fabaceae—*G. max* are characterized by a high content of oxalates [12,13,14].

Oxalic acid can form both water-soluble and insoluble complexes with minerals contained in plants. At a lower pH in the cell sap that is typical of species such as *Oxalis* sp. and *Rumex* sp. (pH ≈ 2), oxalic acid salts are primarily found in the form of water-soluble acid salts, for example, potassium oxalate [14]. This means that oxalates are less likely to accumulate in the body and will not hinder the availability of minerals. Conversely, in plants with a higher pH of the cell sap, such as *Spinacia oleracea* and *Portulaca oleracea* (pH ≈ 6), oxalates are found mainly in the form of insoluble salts—calcium and magnesium oxalate [14]. This, on the one hand, leads to the accumulation of insoluble salts in the body’s tissues, and on the other hand, it interferes with the absorption of important minerals [11]. The adequate daily intake of oxalic acid is between 50 and 200 mg/day, while the minimum lethal dose for adults can reach 5 g, according to some authors [15,16,17]. These plants are rarely consumed raw or only in small quantities. The oxalic acid content can be high in leafy vegetable juice [18]. Usually, they are traditionally subjected to additional culinary processing, such as pickling or heat treatments during cooking or blanching, which reduces their oxalate content. During boiling, a considerable proportion of soluble oxalates is leached into the cooking water, which is typically discarded, resulting in a reduction in their content in the vegetable. In contrast, during pan-cooking, the evaporation of water leads to a concentration of oxalates. Pickling, on the other hand, promotes the extraction of insoluble oxalates due to the acidic environment [13,19]. These techniques leach soluble oxalates and partially destroy the structure of organic acids [17]. Some studies confirm that growing purslane in a greenhouse results in a decrease in total oxalate content in the leaves compared to plants grown in full sunlight. Also, combining purslane with yogurt contributes to a reduction in soluble oxalates in the final product [20].

Oxalate rich plants are often considered minor components of the daily diet, but they can play a significant role in seasonal or specialized diets. This highlights the need for effective control of oxalate content and the implementation of methods for its reduction, particularly when these plants are consumed in larger quantities or incorporated into specific diets.

The determination of oxalate, ascorbic acid, and total organic acid content is traditionally performed by classical chemical methods such as titrimetric, gravimetric, spectrophotometric, and chromatographic methods, which require time, resources, and sample destruction. Near-infrared spectroscopy (NIRS) has been established as a fast, non-destructive, and environmentally friendly approach to the qualitative and quantitative analysis of the chemical composition of plants [21]. The method is based on the specific absorption of infrared light by molecular bonds in organic compounds, which allows the construction of reliable calibration models for predicting chemical composition [22]. More recently, the use of NIR spectroscopy has been reported to determine not only main components such as dry matter, protein, fiber, and oil, but also bioactive compounds in plant materials. Beć et al. [23] published a review of NIR spectroscopy, novel instrumentation, methods for data analysis and interpretation for phytoanalytical applications. An extensive review discussed successful applications of vibration spectroscopy for measurements of nutraceuticals and bio-active components in plants and fruits, the advantages of these techniques, and possibilities using it as an alternative to classical analytical approaches [24]. Publications connected with the quantification of bioactive compounds in food products between 2016 and 2020 were reviewed [25]. The results demonstrate some promise of infrared spectroscopy for the rapid estimation of a wide range of bioactive compounds in food matrices.

To the best of our knowledge, only a few investigations have been reported for the determination of oxalate content in plants and herbal materials. Sperkowska and Bazylak [26] investigated the ability of NIR spectroscopy for determining soluble oxalate content in five single-herbal plant drugs and twenty commercially available multi-herbal products. They reported a coefficient of determination of 0.85 for calibration and validation sets, and a ratio performance to deviation (RPD) of 5.11 for calibration and 4.09 for validation. The authors concluded that the NIRS method could serve as an alternative to the classical “wet chemistry” methods for determining soluble oxalate in plant materials. Gouveia et al. [27] investigated NIRS determination of chemical quality constituents of taro (*Colocasia esculenta* L.) and sweet potato (*Ipomoea batatas* L.) flours. The NIRS prediction of the calcium oxalate content in sweet potato flour was shown to be accurate, with a coefficient of determination for calibration and validation ranging from 0.83 to 0.96, and RPD values of 2.41 for tubers and 2.74 for shoots. The respective results for taro flour were a coefficient of determination ranging from 0.50 to 0.92 and RPD of 2.70 for tubers and 3.58 for shoots.

The present study aims to:

- Estimate the total oxalate content in *P. oleracea* subjected to different types of treatments—fresh, blanched, and pickled.
- Estimate the ability of near-infrared (NIR) spectroscopy for nondestructive determination of total oxalate content, ascorbic acid (vitamin C), and total organic acids in purslane samples.

## 2. Materials and Methods

### 2.1. Purslane Samples

The Purslane samples were collected in 2024 from two floristic regions in Bulgaria—the Thracian lowland and the Sredna Gora mountain. The herbarium specimens from the studied populations have been deposited in the Herbarium of the Agricultural University of Plovdiv (SOA). The locations and geographic coordinates of the populations, along with the accession numbers of the deposited vouchers, are presented in Table 1.

Sampling was carried out three times at 21-day intervals from the same local populations, during the months: July, August, and September, corresponding to Series 1, 2 and 3, respectively. The collected plant mass was washed under running water and divided into three relatively equal parts. One part was measured fresh, the second was blanched, and the third was pickled. For blanching, the stalks were placed in boiling water and boiled for 3 min. After that, the samples were cooled in cold water. The blanched samples were strained and left to drain for 12 h. For pickling, the stalks were left in glass jars with a marinade of salt, sugar, vinegar, and water. The pickling time was 21 days in a refrigerator at 4 °C. Fresh purslane stalks (about 160–200 g), 40 g of sugar, 30 g of salt, 60 mL of 6% vinegar, and water for topping up were used for one 800 mL jar. After the 21st day, the marinade was drained, and the pickled stalks were left to drain for 12 h.

The total number of samples used in the experiment for total oxalate analysis was 36. It is important to note that these are composite samples: for each of the four populations, samples were collected over three consecutive months, with each individual sample consisting of approximately 1.5 kg of material, i.e., including a large number of individual plants and reflecting the average value for the respective population and site. This approach reduces within-sample variability and increases the representativeness of individual observations, which is particularly important when working with spectroscopy of complex plant matrices.

Additional purslane samples from the same floristic regions were collected and used for the determination of ascorbic acid and total organic acids.

### 2.2. Chemical Analysis

The content of total oxalates in fresh, blanched, and pickled samples was determined spectrophotometrically. The method is based on the oxidation reaction of KMnO_4_ with oxalic acid, adapted from Naik et al. [28]. Extraction of oxalate was made with diluted hydrochloric acid. A dry and ground sample (0.5 g) was placed in a 50 mL capacity volumetric beaker with 30 mL of 0.25 N HCl and heated for 15 min at 100 °C. Then, the sample was filtered, and the volume was completed to 50 mL with 0.25 N HCl. Standard oxalic acid solutions were prepared for calibration with concentrations ranging from 0.1 to 0.9 mg/mL. For the determination of oxalic acid, 25 μL of the extract or a blank reagent (water only) was mixed with 125 μL of 2 N H_2_SO_4_ and 50 μL of 0.003 KMnO_4_, and the mixtures were left for 10 min at room temperature. The light absorption at a wavelength of 528 nm was measured.

Vitamin C content and total organic acids were tested from fresh purslane samples. For the aqueous extract, the above-ground parts of *P. oleracea* L. were used, which were cleaned and chopped, distilled water was added in a ratio of 1:1, and then the samples were blended. The extract was filtered and used to determine the content of vitamin C according to the Tillmans method [29] and for the titrimetric determination of total organic acids.

The nutritional and mineral composition of *P. oleracea* from the same experimental fields was comprehensively analyzed in our previous work [9]. Therefore, in the present investigation, we focused specifically on oxalate determination and NIR spectroscopy application, avoiding data duplication.

### 2.3. NIR Spectral Measurements and Spectral Data Processing

The NIR spectra of the tested purslane samples were measured using a NIR Quest 512 spectrometer (Ocean Optics, Inc., Orlando, FL, USA) in the range of 900–1700 nm with a reflection fiber-optics probe. The probe was fixed in the reflection holder to position it perpendicular to the measured samples and at a constant distance, ensuring uniform measurement conditions. The samples were measured as fresh and as the blanched and pickled samples after drainage. The measured samples were placed in Petri dishes, and three dishes of each sample were prepared. Several measurements were taken at different points on the surface of the samples and averaged to minimize any potential effects of variation within the samples.

A Pirouette 4.5 (Infometrix, Inc., Bothell, WA, USA) was used for performing spectral data processing. Partial least squares (PLS) regression was used for the quantitative determination. The calibration equations for each parameter were developed using second derivative spectral data transformation and validated with leave-one-out cross-validation.

PLS regression was used to develop equations for determining total oxalic acid in a separate group of fresh, blanched, and pickled samples, and a group of all purslane samples. The calibration equations for each parameter were developed and validated with leave-one-out cross-validation. The leave-one-out method is recommended when a few samples are used to build the calibration equations. One sample from the calibration dataset was left out, and an equation was developed with the remaining samples. This equation was used to predict the tested parameter for the omitted sample. This procedure was repeated until each sample was used once as a validation sample, and the correlation coefficient between predicted and reference values, and the standard error of cross-validation (SECV) was calculated. The prediction capacity of each calibration equation was evaluated using statistical parameters from the calibration procedure: R—multiple correlation coefficient between reference values and NIR predicted values, standard error of calibration (SEC) and SECV.

Additionally, so-called aquagrams were calculated. An aquagram is a radar chart with coordinates related to wavelengths, corresponding to absorption of free water, and specific water configurations such as dimers, trimers, solvation shells, etc., and named water matrix coordinates [30]. The values for aquagram ${Aq}_{\lambda }$ are calculated using the following equation:

$$Aq_{\lambda }=\frac{A_{\lambda }-\mu_{\lambda }}{\sigma_{\lambda }}$$

where $A_{\lambda }$ is the absorbance at a wavelength λ after multiplicative scatter correction (MSC) transformation of spectral data, $\mu_{\lambda }$ is the mean value of all spectra, and $\sigma_{\lambda }$ is the standard deviation of all spectra at a wavelength λ, respectively. Aquagrams were calculated using the new 19 water matrix coordinates proposed by Vitalis et al. [31].

### 2.4. Statistical Data Analysis

Statistical data analysis includes obtaining the main descriptive statistics (mean values $\left(\bar{x}\right)$ and standard deviations (SD) for the observed parameter total oxalate content g/100 g dry matter (DM) in *P. oleracea*. The Shapiro–Wilk test was used for verification of the normal distribution of the samples. Univariate data analysis with post hoc multiple comparisons at *p*-value < 0.05 was applied to calculate the significant differences between the fresh, blanched, and pickled samples. Depending on Levene’s test of equality of error variances, the Tukey or the Dunnett T3 test was used. The data were processed with the IBM SPSS Statistics 26.0 package.

## 3. Results and Discussion

### 3.1. Total Oxalate Content of Purslane Samples

The results presented in Table 2 reflect the average values of four independent replicates for each series and treatment type of the study, respectively—Series 1 (July), Series 2 (August) and Series 3 (September). The analysis aimed to evaluate the effectiveness of the applied treatments in terms of oxalate reduction, with the fresh samples serving as a control group.

The Shapiro–Wilk test showed that the data for each series and type of processing (fresh, blanched, and pickled) are normally distributed and, despite the small number of samples, provides grounds for using parametric methods of analysis. (Table 2)

The results presented in Table 3 show that the total oxalate content of fresh samples varied within wider limits than that of blanched and pickled samples—33.38 ± 3.85 (Series 2) and 61.84 ± 17.99 g/100 g DM (Series 1). It is important to note that the oxalate values do not represent an independent fraction, but are partially associated with minerals such as calcium, magnesium, iron, and potassium. Oxalic acid occurs in plants as insoluble mineral salts (CaC_2_O_4_, MgC_2_O_4_, Fe(C_2_O_4_)_3_), and soluble salts (Na_2_C_2_O_4_, and K_2_C_2_O_4_), meaning that part of the mineral content is included in the reported values [32]. The high amounts and variations in these minerals in purslane [9] may explain the seemingly high total oxalate values in fresh samples, as well as the significant differences between series and treatments. In addition, a larger standard deviation was observed in Series 1. These features of the variability between series and within Series 1 could be explained by the fact that oxalates in plants are terminal metabolites, the amount of which is variable and depends on the cultivar, growth stage, season, soil conditions, harvest time, and many other factors [33,34,35,36]. In line with this, the overall chemical composition of purslane from these populations has been analyzed in a previous study [9], confirming that such variability is inherent to the species. In the present work, new samples collected the following year were subjected to culinary treatments (blanching and pickling), with the focus placed exclusively on oxalate content and its assessment by NIR spectroscopy, so a full compositional analysis was not repeated. Their levels increased as the growing season progressed [37]. Furthermore, oxalates accumulate unevenly in different parts of the plants, being highest in the leaves compared to buds, stems, or roots [38,39]. Environmental factors such as light intensity and soil moisture also play a role. A study by Kitchen et al. [40] indicated that oxalate concentration in spinach was significantly higher in plants exposed to 12 h of light than in plants grown in shade, suggesting that light is involved in the synthesis of oxalic acid. Oxalates also accumulate under water stress, further supporting the hypothesis of environmental significance [41]. Similar results to ours for the control group in Series 2 and 3 (33.38 ± 3.85 and 34.60 ± 4.91 g/100 g DM) were published by Souza et al. [42], who reported 33.21 ± 0.54 (whole plant) and 32.47 ± 0.53 g/100 g DM (leaves). In comparison, spinach had an oxalate content of 40.11 ± 1.16 g/100 g DM. The negligible effect of blanching in Series 3 (only a 0.69% reduction) can be explained by the higher content of insoluble oxalates in these samples, while blanching removes only the soluble ones [10,13]. Differences in mineral composition (Ca, Mg, Fe, K) in purslane [9] may also influence the formation of insoluble oxalate salts, increasing their resistance to thermal processing. Scientific sources indicate that under such conditions, insoluble oxalates remain or even increase proportionally during thermal treatment [10,12,13]. Conversely, pickling, due to its acidity and prolonged exposure, reduces the total oxalate content in all series, likely through reaction or extraction of the insoluble forms [43,44]. Our results show that both blanching and pickling significantly reduced the total oxalate content of purslane. Pickling was more effective, with reductions ranging from 47.07% (Series 3) to 83.06% (Series 1), while blanched samples showed reductions ranging from 0.69% (Series 3) to 69.16% (Series 1). The trends observed in our study are supported by other publications that also found that heat treatment and acidic environments can lead to significant oxalate losses in plant products. Savage et al. [10], for example, reported a reduction in total oxalate after heat treatment of various leafy greens and root vegetables, noting that soluble oxalate was significantly reduced compared to insoluble oxalate. An important consideration is that longer blanching times and higher temperatures enhance the reduction in soluble oxalates but can also lead to losses of water-soluble vitamins and minerals. Optimizing blanching conditions is therefore essential to balance oxalate reduction with the preservation of nutritional quality [42,43,44,45]. Regarding the oxalate content after pickling, the results are as follows: Series 1—10.48 ± 3.46, Series 2—17.40 ± 5.44 and Series 3—18.31 ± 4.65 g/100 g (DM), close to our values, but with a different pickling method reported by Waleed et al. [43], namely—14.39%.

Statistically significant differences in the total oxalate content were reported for all three series between fresh and processed samples (Table 3). Only in Series 3 there was no difference in the total oxalate content in fresh and blanched samples, but they differed significantly from the oxalates in pickled purslane samples. The coefficients of determination (R^2^) for the three series ranged from 0.690 to 0.835 (Table 3), i.e., from 69.0% to 83.5% of the variation in the total oxalate content was due to the influence of the different treatments (blanching or pickling).

Although the specific values vary depending on the preparation methods used (blanching and pickling) or extraction, analytical approach, and starting material (fresh or dried), the general conclusion is that culinary treatments represent an effective strategy for reducing potentially undesirable compounds such as oxalates.

### 3.2. NIR Spectroscopy Determination of Total Oxalate Content

The near-infrared spectra of purslane samples were measured as fresh ones, as blanched and pickled samples after drainage, without drying and grinding. Because of this, the concentration of total oxalate content was recalculated on a fresh basis. The moisture content varied from 88.4 to 92.4% for fresh, from 87.8 to 93.7% for blanched, and from 75.7 to 83.8% for picked samples, respectively. The range of oxalate content after recalculation was from 2.52 to 7.97 g/100 g for fresh samples, from 0.73 to 4.35 g/100 g for blanched samples, and from 1.58 to 5.05 g/100 g for pickled samples. These values were used in the analysis of spectral characteristics and creation equations for quantitative determination.

Oxalic acid is a dicarboxylic acid, H_2_C_2_O_4_, containing two carboxyl groups. These functional groups exhibit weak overtone and combination bands in the near-infrared range. Therefore, we can expect that the spectra of purslane samples with different oxalate contents will show some differences.

**Figure 1:**
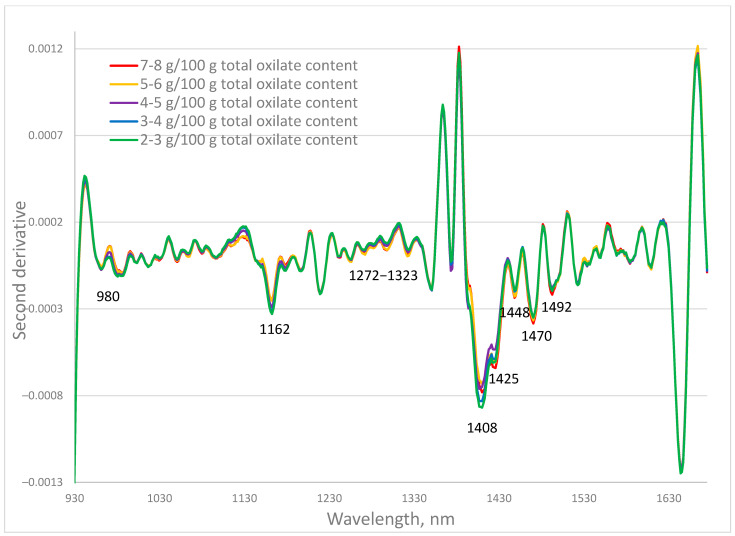
Second derivative spectra of fresh purslane (*Portulaca oleracea* L.) samples with different total oxalate content.

The investigated fresh samples have a wide range of oxalate content. The second derivative spectra of the *P. oleracea* fresh leaves containing total oxalates in the ranges 2–3; 3–4; 4–5; 5–6; and 7–8 g/100 g total oxalate were averaged and presented in Figure 1. The second derivative transformation allowed separation of overlapping peaks that are hard to distinguish in the original spectrum and makes small or hidden peaks more visible, especially useful for detecting minor compounds. The largest differences are observed at 980 nm, 1162 nm, and the range of 1272–1323 nm, as well as at 1408 nm, 1425 nm, 1448 nm, 1470 nm, and 1492 nm. Absorption at these wavelengths was related mainly to the C=O group at 1162 and 1448 nm, and O–H at 980, 1408, 1425, and 1470 nm.

**Figure 2:**
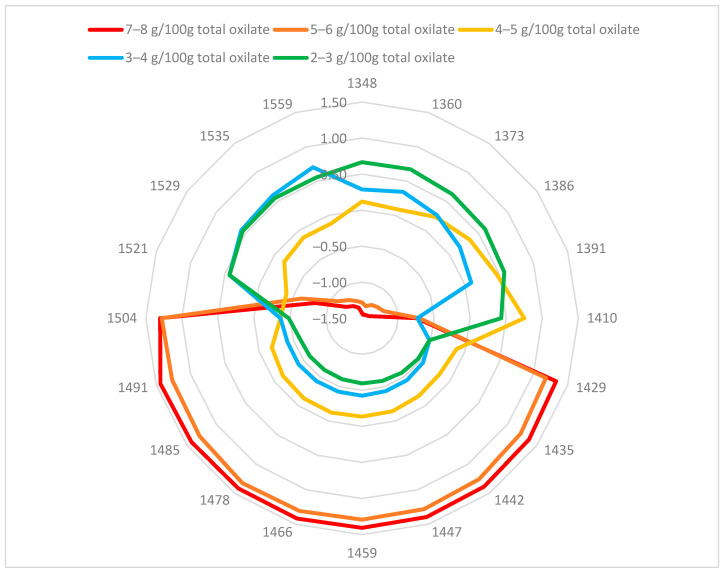
Aquagram of fresh purslane samples with different oxalate content.

Oxalic acid can form hydrogen bonds with water molecules. In aqueous solutions, oxalate ions also react with water to produce hydrogen oxalate and hydroxide ions. A change in oxalic acid and oxalate content would also change the structure of water in the purslane plant cells. To reveal the changes in the water molecular matrix caused by differences in oxalate content, the NIR spectra of fresh purslane were studied using the aquaphotomics approach. The aquagram for fresh purslane samples with different total oxalate content is presented in Figure 2. The spectral patterns depended on the total oxalate content. Aquagrams of samples with total oxalate content between 5 and 8 mg/100 g fresh weight (FW) in fresh leaves were different from those of lower oxalate content.

Aquagram values for samples with higher oxalate content increased in 1429–1503 nm. The water structure in this region corresponded to the water solvation shell (aquagram values at 1447 and 1454 nm), water dimer at 1441 nm, and water molecules with two, three, or four hydrogen bonds (aquagram values 1465, 1478, and 1485 nm) and strongly bound water at 1503 nm. Generally, the aquagram coefficients for samples with oxalate content lower than 5 mg/100 g were lower than those for samples with higher oxalate content. The maximum values were in the ranges of 1349–1410 nm and 1521–1559 nm. The region of 1521–1559 nm is primarily associated with structural water, resulting from the intermolecular interactions of water with cellulose or protein [46]. The area 1349–1410 nm corresponds mainly to free or quasi-free water and solvation shells around ions. Therefore, increasing the amount of oxalates in the analyzed purslane plants leads to a decrease in free water in them and an increase in water molecules forming two, three, or four hydrogen bonds.

The correlation between aquagram coefficients and total oxalate content of fresh purslane samples was higher than 0.7 (from 0.70 to 0.83), except for those at 1410 and 1521 nm. Correlation coefficients were positive in the range 1429–1504 nm and negative in the ranges 1348–1410 nm and 1521–1559 nm.

The aquagrams clearly showed changes in the ratio of free and bound water, as well as the number of hydrogen bonds between water molecules, in fresh purslane samples with varying oxalate content. Aquaphotomics analysis could be used as an additional approach in the analysis of purslane samples.

### 3.3. NIR Spectroscopy Quantitative Determination of Total Oxalate in Purslane Samples

The near-infrared spectra of purslane samples contain information about all the chemical components in them. Oxalates are not just one chemical substance. Information about variations in oxalate content in the near-infrared spectral characteristics was observed at different wavelengths, as was seen in Figure 1. This requires the use of multivariate statistical methods, such as PLS regression, to quantify the oxalate content in the analyzed purslane samples. PLS create new latent variables that maximize the covariance between the absorption at different wavelengths and the determined parameter. The first factor describes the maximum part of the variations in both the spectral characteristics and the estimated parameter. The second describes a maximum part of the remaining variations, and so on. Once these factors are created, a multiple linear regression equation is applied using these new factors as predictors. The optimal number of factors in the equation is the one that resulted in a minimal standard error of cross-validation.

**Figure 3:**
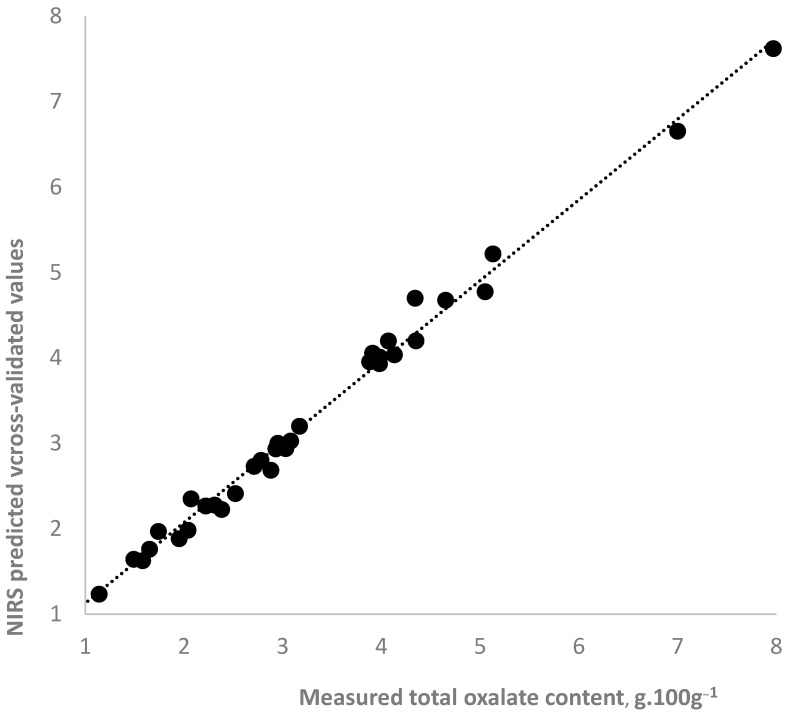
NIR spectroscopy prediction of total oxalate content in wet purslane samples using PLS equation for all samples.

Table 4 presents the statistics for the NIR spectroscopy equations used for predicting total oxalates in fresh, blanched, pickled, and all analyzed purslane samples. A graphical illustration of the accuracy of the NIR spectroscopy prediction of the total oxalate content in all samples is presented in Figure 3. The determination accuracy was very high. Calibration and cross-validated correlation coefficients ranged from 0.97 to 0.99, indicating a strong dependence between the oxalate content and the spectral characteristics of purslane samples. As can be expected, the accuracy of determination in the individual equations for fresh, blanched, and pickled samples is better compared to the equation for the combined group of all samples.

In near-infrared spectroscopy, a statistical parameter RPD (ratio performance to deviation) is used to evaluate the performance and accuracy of NIRS calibration equations. An RPD value is a ratio of the standard deviation of the constituent’s values in the sample set to the standard error of prediction or cross-validation. It takes into account both the range in the parameter being tested and the equation’s prediction error. RPD values bigger than three indicate an accurate and reliable equation. The RPD value for the equation for all samples was 4.04, which proved that the NIRS equation can accurately predict total oxalate in purslane samples. No difference was observed in the accuracy of determining the oxalate content in the fresh and treated purslane samples using the equation based on the spectra for all samples. The RPD for the individual equations for fresh, blanched, and pickled samples was bigger than 10.

In summary, the near-infrared spectroscopy methods presented in this study can be used to fast and accurately estimate the total oxalate content in fresh, blanched, and pickled purslane samples, measuring directly and nondestructively fresh plants or culinary processing purslane samples.

### 3.4. NIR Spectroscopy Determination of Ascorbic Acid and Total Organic Acids Content

Purslane contains several organic acids such as malic acid, citric acid, oxalic acid, and ascorbic acid, which contribute to its health benefits [47]. Purslane is especially high in ascorbic acid, and it is considered one of the richest plant sources among leafy green vegetables. These values can vary depending on plant maturity, growing conditions, and specific cultivar. Therefore, their rapid and non-destructive determination is important.

The range of ascorbic acid in the fresh samples investigated was from 8.86 to 30.50 mg·100 g^−1^ and the mean values of 16.27 mg·100 g^−1^ (Table 5). The respective values of range for organic acids were from 0.14 to 0.37 g·100 g^−1^ with an average value of 0.22 g·100 g^−1^.

**Figure 4:**
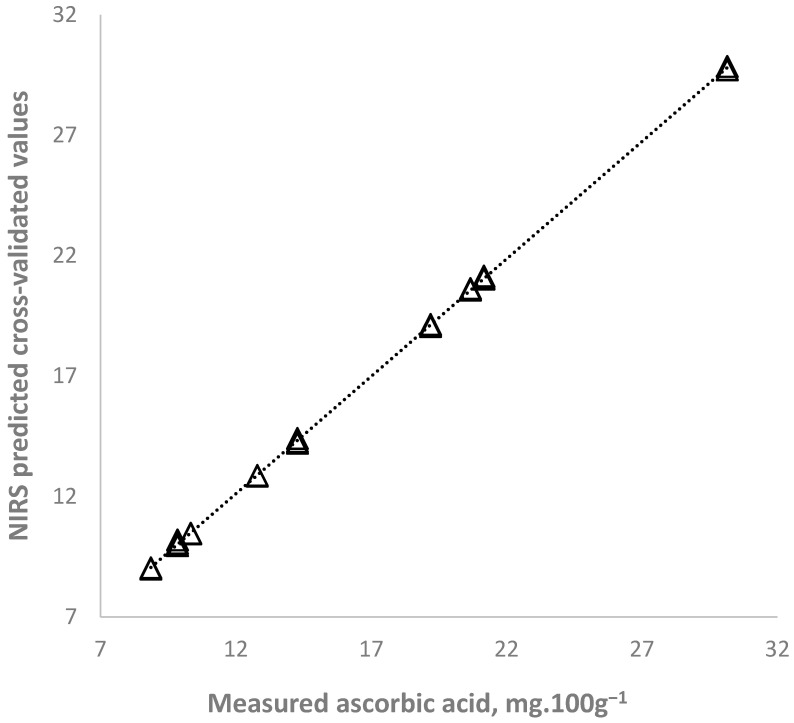
NIR spectroscopy prediction of ascorbic acid content in purslane samples.

The NIR spectroscopy determination accuracy was very high—calibration and cross-validated correlation coefficients were 0.99, indicating a strong dependence between the ascorbic acids and total organic acids and the spectral characteristics of purslane samples (Table 6). Graphical relationships between reference values and predicted values of ascorbic acid content are presented in Figure 4. The RPD values of the equations for determination of both parameters was bigger than 10.

Organic acids and ascorbic acid have been identified and quantified in purslane but not specifically using near-infrared spectroscopy in the available scientific literature. There were several publications reporting successful application of NIR spectroscopy for determination of ascorbic acid and organic acids in some fruits and vegetables, such as bell pepper [48] acerola fruit [49], and oranges [50]. Our results confirmed that NIR spectroscopy would be a novel and promising direction, especially for rapid nondestructive screening.

## 4. Conclusions

The content of total oxalate, organic acids, and ascorbic acid in the analyzed fresh purslane samples varies widely during a growing season. Culinary treatments (blanching and pickling) reduced oxalate content. Pickling reduced the oxalate content more significantly compared to blanching.

There were changes in the NIR spectra of purslane samples, connected with their oxalate content. The aquaphotomic approach revealed changes in the ratio of free and bound water, as well as the number of hydrogen bonds between water molecules, in fresh purslane samples with varying oxalate content. The determination accuracy of the obtained NIR equations for the determination of total oxalate, organic acids, and ascorbic acid content was very high.

NIR spectroscopy could be used as an alternative to traditional analytical methods for the analysis of purslane samples directly in the plant matrix without complicated sample preparation or using toxic reagents. NIR spectroscopy would be used for rapid screening of purslane plants according to their oxalate, organic acids, and ascorbic acid content.

Although the number of samples analyzed was limited, the obtained calibration and validation results demonstrated a strong relationship between NIR spectral data and total oxalate content. Expanding the dataset with additional samples from different populations and growing conditions in future research will further confirm the robustness and broader applicability of the developed NIR model.

## Figures and Tables

**Table 1:** Studied populations of purslane (*Portulaca oleracea* L.).

Voucher Speciments	Locations and Geographic Coordinates			
	Region	Longitude	Latitude	Altitude
SOA 063610	Thracian lowland, town ofStara Zagora, in the Kolyo Ganchev neighborhood	42°24′06.8′′ N	25°38′10.8′′ E	196
SOA 063611	Thracian lowland, village of Madzherito	42°21′48.2′′ N	25°38′43.7′′ E	160
SOA 063612	Sredna gora mountain, village of Malka Vereya	42°25′07.9′′ N	25°33′22.7′′ E	353
SOA 063613	Sredna gora mountain, village of Pryaporets	42°27′51.4′′ N	25°31′57.2′′ E	466

**Table 2:** Tests of Normal distribution of the total oxalate content g/100 g in Fresh, Blanched and Pickled samples of *P. oleracea* L.

Tests of Normality								
Shapiro–Wilk			Series 1		Series 2		Series 3	
Total oxalate content g/100 g (DM)	Treatment	df	Statistic	Sig.	Statistic	Sig.	Statistic	Sig.
	Fresh, (control)	4	0.960	0.778	0.998	0.992	0.861	0.262
	Blanched	4	0.873	0.308	0.886	0.365	0.874	0.314
	Pickled	4	0.760	0.051	0.925	0.563	0.964	0.804

Lilliefors Significance Correction.

**Table 3:** Effect of blanching and pickling on total oxalate content in *P. oleracea* L.

Treatment	Total Oxalate Content (g/100 g DM)		
	Series 1	Series 2	Series 3
Fresh, (control)	61.84 ± 17.99 ^a^	33.38 ± 3.85 ^a^	34.60 ± 4.91 ^a^
Blanched	19.07 ± 7.90 ^a^ (−69.16)	22.29 ± 6.00 ^a^ (−33.22)	34.36 ± 1.88 ^b^ (−0.69)
Pickled	10.48 ± 3.46 ^a^ (−83.06)	17.40 ± 5.44 ^a^ (−47.86)	18.31 ± 4.65 ^ab^ (−47.07)
Levene’s Test	0.003	0.769	0.225
Sig.; R^2^	0.001; 0.835	0.005; 0.690	0.001; 0.825

The values in parentheses indicate the percentage by which the oxalate content has decreased (−) or increased (+). Each value is the mean of four replications of different batches of plants ± SD; DM—dry matter; ^a,b^ Same superscripts within the columns represent significant differences at the level of significance *p* < 0.05; post hoc tests Tukey or Dunnett T3 depend on Levene’s test of equality of error variances.

**Table 4:** Statistical parameters of NIR models for the determination of total oxalate in purslane samples using PLS regression.

Total Oxalates, g 100 g^−1^	PLS Factors	SECV	r_cval_	SEC	r_cal_
All samples	4	0.388	0.97	0.383	0.97
Fresh samples	4	0.017	0.99	0.014	0.99
Blanched samples	5	0.012	0.99	0.010	0.99
Pickled samples	8	0.029	0.99	0.01	0.99

r_cval_ and r_cal_—multiple correlation coefficients between reference values and NIR predicted values for cross-validation and calibration, respectively; SEC—standard error of calibration, SECV—standard errors of cross-validation.

**Table 5:** Content of ascorbic acid and organic acids content in purslane samples.

Parameter	Min	Max	Average	SD
Ascorbic acid, mg·100 g^−1^ FW	8.86	30.50	16.37	6.34
Total organic acids, g·100 g^−1^ FW	0.14	0.37	0.22	0.08

**Table 6:** Statistical parameters of NIR models for the determination of ascorbic acid and organic acid in purslane samples using PLS regression.

	PLS Factors	SECV	r_cval_	SEC	r_cal_
Ascorbic acid, mg·100 g^−1^ FW	3	0.173	0.99	0.042	0.99
Total organic acids, g·100 g^−1^ FW	4	0.0034	0.99	0.0004	0.99

r_cval_ and r_cal_—multiple correlation coefficients between reference values and NIR predicted values for cross-validation and calibration, respectively; SEC—standard error of calibration, SECV—standard errors of cross-validation.

## Data Availability

The original contributions presented in this study are included in the article. Further inquiries can be directed to the corresponding author.

## Abbreviations

NIR	near-infrared
NIRS	near-infrared spectroscopy
DM	dry matter
FW	fresh weight
SD	standard deviations
RPD	ratio performance to deviation
MSC	multiplicative scatter correction
PLS	partial least squares
SECV	standard error of cross-validation
SEC	standard error of calibration
